# Risk Perception and Coping Behavior of Construction Workers on Occupational Health Risks—A Case Study of Nanjing, China

**DOI:** 10.3390/ijerph18137040

**Published:** 2021-07-01

**Authors:** Hui Liu, Jie Li, Hongyang Li, He Li, Peng Mao, Jingfeng Yuan

**Affiliations:** 1 Department of Construction Management, College of Civil Engineering, Nanjing Forestry University, Nanjing 210037, China; liuhui@njfu.edu.cn; 2 School of Civil and Transportation Engineering, Shenzhen University, Shenzhen 518060, China; lijie20181@email.szu.edu.cn; 3 Business School, Hohai University, Nanjing 211100, China; lihy@hhu.edu.cn; 4 School of Civil Engineering and Transportation, South China University of Technology, Guangzhou 510641, China; 5 State Key Laboratory of Subtropical Building Science, South China University of Technology, Guangzhou 510641, China; 6 College of Civil Engineering, Hunan University, Changsha 410082, China; lihe@hnu.edu.cn; 7 Department of Construction and Real Estate, School of Civil Engineering, Southeast University, Nanjing 210096, China; jingfeng-yuan@seu.edu.cn

**Keywords:** construction workers, occupational health, health risks, risk perception, risk coping behavior

## Abstract

To reduce harm caused by occupational health risks of construction workers exposed to working environments, especially those for interior decoration, it is crucial for them to actively recognize and prevent these risks. Therefore, how to improve their occupational health risks perception and regulate their coping behaviors should be of great concern. However, most prior studies target construction worker safety, and little research focuses on risk analysis from the psychological level of workers. Hence, construction workers’ occupational health risk perception level and coping behavior level in Nanjing and the influencing factors were analyzed through statistical analysis with 341 valid questionnaires. Bootstrapping was applied to test the mediating effects of risk perception on the proposed factors and coping behaviors. This study revealed that construction workers have a high-level of occupational health risk perception, yet low-level coping behavior. Gender, age, education level, and unit qualification cause differences in individual risk perception level. Personal knowledge and group effects significantly affect the level of risk perception, which subsequently affect coping behavior. Education level, monthly income, and personal knowledge influence the coping behavior through risk perception. Recommendations were put forward for risk perception and coping behavior improvement from the perspectives of construction workers themselves, enterprises, and governments. This study sheds new light for research areas of occupational health and risk management and provides beneficial practice for improving construction workers’ responses to occupational health risks.

## 1. Introduction

Safety has always been a prominent problem in the construction industry and has been the concern of many scholars [1,2,3]. Despite the adoption of various methods, such as the establishment of construction site safety management systems [4,5], real-time safety monitoring [6,7], and identification of key factors affecting workers’ safety behavior [8,9], the safety situation of the construction industry has been significantly improved. However, construction workers need to face not only safety issues, but also occupational health issues [10]. The construction industry is considered to be an industry with a high incidence of occupational hazards because of its numerous and complex types of occupational hazards, various construction materials containing harmful substances, and diversified construction sites [11,12,13]. Construction workers are exposed to such a harsh working environment every day, and their health is bound to be threatened by the toxic and harmful substances produced in the construction process [14]. However, at present, from the perspective of construction workers’ attitudes to occupational health protection and their enthusiasm to take protective actions, they do not pay enough attention to the occupational health risks they face [15]. In addition, serious health problems easily lead to work efficiency reduction, construction period delay, cost increases, and other issues [16,17,18]. If occupational health problems of construction workers cannot be paid enough attention and effectively solved for a long time, it will affect the long-term healthy development of the construction industry. Therefore, the occupational health of construction workers is a subject of practical significance. Government has spent a great deal of time and effort attempting to evolve legislation, regulations, and rules to help reduce safety incidents and occupational health hazards so as to promote the sustainable and healthy development of the construction industry. In China, construction safety practices are regulated by the Ministry of Housing and Urban–Rural Development (MOHURD), which provides strict rules and regulations to enforce safety and health standards on the job site. For example, the “Notice on Further Improving the Working and Living Environment of Construction Peasants and Effectively Protecting the Occupational Health of Migrant Workers” promulgated in 2006 pointed out that to effectively protect the occupational health of migrant workers requires actively carrying out vocational skills and safety education and training for migrant workers in the construction industry. In addition, the government has also issued a number of standards, such as the Standard of Environment and Sanitation of Construction Site (JGJ 146-2004), and the Occupational Health and Safety Management Systems-Requirements (OHSAS 18001: 2007, IDT), to enable the construction industry to control its occupational health and safety risks and improve its occupational health and safety performance. However, these health and safety regulations and policies alone cannot reduce the incidence of accidents and reduce occupational health harm. Unless workers themselves realize the importance of occupational health and take active actions to incorporate these rules into their daily activities, they cannot effectively protect their own safety and health [19]. Thus, it is worth stating that occupational health protection is a critical issue that should be human oriented. Specifically, all safety and health measures must be based on human involvement [20]; thus, workers’ perceptions and behaviors of occupational health risks are crucial factors to ensure the formulation, adoption, and maintenance of strategies. Moreover, the behavior taken by individuals largely depends on individual’s cognition and judgment of the event itself [21]. Occupational health risk perception is a long-term process for workers to recognize and understand occupational health risks and use relevant knowledge and experience to deal with these risks. However, the research on risk perception and coping behavior of construction workers mostly focuses on the safety perspective to explore the influencing factors of risk perception and the effectiveness of coping behavior. What remains largely unknown is the occupational health risk perception and coping behavior of construction workers, not to mention the relationship between them.

Significantly, in the group of construction workers, there is a special group—construction workers for interior decoration, which refers to plumbers who connect and repair water pipes, electricians who design household circuits, carpenters who make furniture, painters who paint walls, bricklayers who build walls, and other workers engaged in different types of work during the interior decoration process. Due to the particularity of their work, such groups have more complex occupational health problems than ordinary construction workers [22]. Their working space is relatively narrow, even closed, and they often conduct their work in such environments polluted by construction pollutants for a long time, so they tend to suffer from a variety of pollutants at high concentrations [23,24]. Studies have shown that the working environment of construction workers for interior decoration is not ideal, and the concentration of harmful factors in various positions exceeds the national health limit [25,26]. In addition, the education level of such groups is generally low, and they are lacking in knowledge about construction risk factors and personal protective measures, and compared with ordinary construction workers, their working sites are generally not managed by managers, so the health problems faced by these workers are more serious [22,27]. However, the health impact of indoor environmental risk after construction on occupants has been paid much attention, while the health damage caused by indoor harmful substances to construction workers in the construction process is ignored at present. Therefore, the occupational health of construction workers for interior decoration is the target in this paper to discuss occupational health risk perception and coping behavior of construction workers, and to provide a new research perspective for the occupational health of construction workers.

Consequently, this research aims to examine the occupational health risks of construction workers and to analyze their occupational health risks from the perspective of workers’ themselves. Five specific objectives are as follows: (1) what levels are construction workers’ occupational health risk perceptions and coping behaviors reached in Nanjing, China; (2) whether the individual characteristics affect construction workers’ risk perceptions differently; (3) based on the developed model, whether the mentioned factors affect risk perception and coping behaviors; (4) whether the mediating effect of occupational health risk perception exists; and (5) what suggestions could be proposed for construction workers’ risk perceptions and coping behaviors improvement to facilitate the sustainable development of construction industry. The findings of this research are expected to contribute to the existing body of knowledge in terms of occupational health and risk perception. Furthermore, this study provides valuable implications for guiding construction workers to better prevent their occupational risks and enhance their coping capacity, as well as providing a theoretical foundation for the government and the construction enterprises to formulate rules and regulations.

## 2. Literature Review

### 2.1. Occupational Health Risk

Occupational health risk refers to the possibility of adverse impacts on the health of employees exposed to occupational health hazards in a specific period of time or environment [19,28,29]. At present, there is some research on occupational health risks for construction workers, but little for construction workers for interior decoration.

The occupational disease rate of construction workers is higher than the average occupational disease level [30]; it is thus extremely important to focus on the occupational health risks of construction workers. In the existing research on the occupational health risk of construction workers, it is mainly concentrated on the construction of workers’ occupational health damage assessment models, the health damage caused by specific pollutants such as noise and dust to construction workers, and the establishment of health risk assessment systems for construction sites. For instance, Li et al. [31] built a health damage assessment model to quantify the degree of hearing damage caused by occupational noise during construction activities. Tong et al. [32] established a probabilistic risk assessment model when investigating the impact of construction dust on the health of construction workers. Chen et al. [33] constructed a health risk assessment system to reveal the health risk characteristics of dust in tunnel construction, which is suitable for the characteristics of tunnel construction. The innovation of the system is that it gives economic significance to health risk, reduces the cost of risk, and improves the accuracy of assessment. In addition, other scholars have proposed some targeted countermeasures based on the identified hazards in construction projects [34]. Khan et al. [35] pointed out that chemical hazards are the biggest hazards faced by Pakistan’s construction industry, followed by fire hazards. Construction companies should actively carry out occupational health and safety training for employees [36,37], and allocate sufficient budgets [20,38], especially for personal protective equipment [20], to protect workers from accidents. Moreover, it is not that employees do not correctly implement safety regulations, but that most construction companies do not have appropriate safety rules [38,39]. However, in construction projects, more attention is paid to safety than occupational health. For workers, the most intuitive injury they perceive is a safety accident, such as a fall from a high altitude and other casualties. Such accidents tend to cause injuries to workers in a short period of time, while health is more concerned with diseases, which cannot be detected by workers in a short period of time. For example, due to working in an environment with high formaldehyde concentrations for a long time, the nervous system, immune system, and digestive system of workers will be damaged to varying degrees [40]. It is precisely because such risks have a long-term and cumulative effect on workers’ health that workers often tend to ignore the impact of various risks on their health at work. Therefore, workers and managers need to change the means of their perception of occupational health [41].

It can be observed that although the existing research has accumulated some achievements in aspects of occupational health risk, research based on the perspectives of construction workers is still insufficient.

### 2.2. Risk Perception 

The research on risk perception can be traced back to the 1960s; Starr [42] found that risk acceptability needs to consider people’s subjective scale. Then, Slovic et al. [43] used a psychological measurement paradigm model to measure different dimensions of risk perception. In their research, risk perception represents an individual’s cognition and understanding of various objective risks existing in the outside world, and the influence of an individual’s experience gained from intuitive judgment and subjective feelings on perception are emphasized [44]. Later, many other scholars redefined risk perception. Desai [45] posited that risk perception refers to the process of individual assessment of situations including risk, mainly involving the description of situations, the estimation of risk controllability, and the probability of occurrence. In view of the definitions of risk perception mentioned above, it is specially studied as an individual cognitive mechanism. In this mechanism, the individual has nothing to do with the social system. However, the theory of the individual level cannot explain how the perception of risk changes between or within communities [46]. Therefore, Leiserowitz [47] broke through the individual level, redefined the risk perception, and creatively proposed that risk perception has surpassed the individual, which is a kind of social and cultural construction reflecting the value, representation, history, and ideology of various factors in the social system. According to current research, the main factors affecting risk perception are individual differences, expectation level, the influence of information, the nature of risk characteristics, voluntary degree, and education level [48,49,50]. To sum up, the common views in defining risk perception include the following: people not only need to know the existence of risk, but also need to feel that they are at risk in order to take protective measures, and individual risk perception will be affected by various factors in the social system.

The research concept of risk perception mainly includes public crisis events such as emergencies or natural disasters [50], even though some scholars have introduced risk perception into the field of health to explore individual health. Jeong [51] investigated the influence of health literacy and health risk perception on the health behavior of the elderly in South Korea. The results showed that there was a negative correlation between health risk perception and health behavior, and a positive correlation between health literacy and health behavior. Renner [52] discussed the transition process from understanding risk to perceiving risk, the change of health risk perception over time, and its impact on health-related behaviors. Many studies have shown that health risk perception plays an important role in understanding and predicting health diseases [53,54]. Although risk perception has accumulated many outstanding achievements in the field of health, there are few studies on the health risk perception of specific groups, such as construction workers. This is due to the diversity, risk, and many harmful factors of construction site activities; scholars pay more attention to the safety of construction workers [55,56,57]. It is undeniable that the ability of construction personnel to identify, perceive, and assess risks is a basic skill to maintain the safety of construction sites. Chaswa [58] found that risk perception was significantly associated with age, personal knowledge, and education level when investigating the influencing factors of risk perception of construction workers. Man [59] found that previous studies did not consider the emotional risk perception of construction workers, and personal emotions also affect their perception of risk. Therefore, he developed a psychometrically reasonable tool, namely a construction worker’s risk perception scale, to evaluate the risk perception of construction workers. It can be seen that the construction worker’s perception of safety risks is valuable for determining and eliminating the risks on the construction site. In the research on health risk of construction workers, most of them associate the risk perception of construction workers with their health behaviors and protective measures [60]. Strickland [61] found that although people have a high understanding of work-related health and safety risks, they pay less attention to general health risks. This result proves the effectiveness of education and training. Gürcanlı [62] found that Turkish construction equipment operators who received safety and health training had different perceptions of risk than others. Arezes [63] pointed out that risk perception should be regarded as an important issue in the design and implementation of a hearing protection plan when investigating workers exposed to high noise environments. Hence, it is necessary to study the occupational health risk of construction workers. 

As a result, existing research on risk perception mainly focuses on natural disasters, with low attention to health risks and construction workers. Research on health risk perception mainly discusses the formation of health risk perception and its impact on individual health behavior. Most scholars believe that health risk perception will affect health-related behaviors, but the concept of coping behavior has not been introduced to systematically analyze the relationship between the two. Moreover, most of the research on construction workers focuses on safety issues and targets the measurement paradigm of safety risk perception, and there is no research on the occupational health risk perception of construction workers, let alone construction workers for interior decoration.

### 2.3. Coping Behavior

When people encounter hazards or threats, they will make decisions by considering risk assessment and response assessment, that is, to take actions they deem appropriate [64,65]. Coping is defined as “changing cognitive and behavioral efforts to manage specific external and/or internal needs” [66]. Coping behavior is an activity in which people consciously, purposefully, and flexibly change their emotions, perceptions, behaviors, and environments under pressure and perceived risk; that is, they consciously reduce the risk of threatening events in the environment [66,67]. Therefore, it is not difficult to find that coping behavior usually occurs in a stressful environment and is often the external performance of their own pressure and anxiety. Coping behavior is mainly divided into three types: planned problem-solving, emotional discharge, and positive reappraisal [68]. Planned problem-solving is an individual’s behavior to deal with stressful problems. Emotional discharge refers to the output of unpleasant, angry, and other emotions. Positive reappraisal mainly focuses on whether individuals re-analyze and solve problems in a positive way [66,68].

When reviewing research on coping behavior, it is found that most scholars are committed to exploring the determinants of individual risk coping behavior. By constructing models of coping protection decision-making, risk information searching, and processing, they revealed the behavior decision-making process of individuals in the face of sudden danger [69,70]. Different individuals adopt different coping behaviors [68,71], which may be affected by individual characteristics, social environments, and emotions [72,73,74]. Tamres [75] pointed out that women are more likely to participate in most coping strategies than men, and they are more likely to seek emotional support. Martin [76] evaluated the age, gender, race, and education differences of 35 specific health coping behaviors among three age groups and found that 14 coping behaviors had significant differences in age group, gender, and education level, and the difference of age group had the greatest impact on the acceptance of health problems. Construction workers, who are generally undereducated and predominantly male, are often among the most vulnerable to neglect in the construction organization. Therefore, although coping behavior is the key in the process of individual stress management, few studies have explored the coping behavior of construction workers, and most of them have revealed the results of different types of coping behavior of construction workers from the perspective of safety management. Liang [77] established the coping behavior–pressure–safety model of construction workers and found that different types of coping behaviors have different effects on the pressure of construction workers. Active coping and planned problem solving can directly improve the safety participation of construction workers, and avoidance behavior will cause safety disobedience. However, construction workers face high pressure and complex working environments, which will bring adverse effects on their health. Only paying attention to the occupational safety of construction workers is far from enough. It is positive coping behavior that can reduce the possibility of psychological symptoms of construction workers and ensure their occupational health [78,79].

It can be seen that most studies on coping behavior focus on the influencing factors and different types of coping behaviors. However, less attention has been paid to construction workers’ coping behaviors, and the existing research is mostly from the perspective of safety management, aiming to explore the influence of different types of coping behavior on construction workers’ own stress and safety participation, and few studies focus on the occupational health of construction workers. Construction projects involve a variety of harmful and complex pollutants. Construction workers’ long-term work in such a complex environment is bound to cause indelible harm to their occupational health. Therefore, it is necessary to study the impact of coping behavior on the occupational health of construction workers.

## 3. Hypothesis Development

Individuals have different characteristics, such as gender, age, education level, and so on. These characteristics result in the differences of event perception among individuals and also affect the formation of their risk perception. Therefore, the risk perception level of different people is totally different. For example, Park [80] found that the higher education level, the higher risk perception of IoT services. This can be explained as, the higher the level of education, the greater the understanding of potential IoT risks. Women generally had more awareness of risk than men [81]. In addition, in general, individual life experience will increase with age, resulting in another influential factor of age; workers with more life experience may have a more comprehensive risk perception in the construction process. For example, Blanco et al. [82] believed that with lifelong decision-making experience, older people may be able to rely on experienced strategies to make effective decisions in the real world. However, in previous research, gender, marital status, and age were also confirmed to have very little or no influence on workers’ perceptions of risk. Moreover, Ren et al. [83] pointed out that the public’s income was an important factor influencing risk perception toward a highly protested waste-to-energy facility. In addition, when people work in different units, the units have different health management capabilities methods, as well as levels of employee education. We therefore propose the following hypothesis:

**Hypothesis** **1a** **(H1a).** *Gender contributes to construction workers’ perceptions of occupational health risk.*

**Hypothesis** **1b** **(H1b).** *Age is positively associated with construction workers’ perceptions of occupational health risk.*

**Hypothesis** **1c** **(H1c).** *Marital status contributes to construction workers’ perceptions of occupational health risk.*

**Hypothesis** **1d** **(H1d).** *Education level is positively associated with construction workers’ perceptions of occupational health risk.*

**Hypothesis** **1e** **(H1e).** *Monthly income is positively associated with construction workers’ perceptions of occupational health risk.*

**Hypothesis** **1f** **(H1f).** *Unit qualification is positively associated with construction workers’ perceptions of occupational health risk.*

To study what influences construction workers’ risk perceptions of occupational health risks, both workers’ knowledge degree of the risk and their social environment should be taken into consideration.

On the one hand, since people have different understandings of the possibility of risk, the severity of consequences, and the ability to control a certain event, they will make different cognitive reactions when facing risk. For instance, after summing up the research of other scholars, Visschers et al. [84] pointed out that increasing people’s knowledge through communication and education is most likely to affect their cognition of danger. Aluko et al. [85] argued that knowledge of potential occupational hazards is closely related to the formation of a positive attitude, which will provide information for behavior. Along similar lines, construction workers with more knowledge will lead to a better understanding of the occupational health risks, and as a result, they will have a higher level of risk perception. Hence, the following is hypothesized:

**Hypothesis** **2** **(H2).** *Personal knowledge has a positive impact on occupational health risk perception of construction workers.*

On the other hand, a person is not independent in this society, and he or she will be affected by various subjects of the society and the events. In a corporate context, Namian et al. [86] proposed that workers with high engagement training were able to identify a greater proportion of hazards and therefore perceived safety risks to be relatively high. However, it is worth mentioning that the main people carrying out training are the managers. In addition, the construction workers themselves have a low level of education, so the construction workers alone can hardly achieve a highly engaging training [87]. Zhao et al. [88] revealed that the participation of senior managers played a positive role in the implementation of risk management. Important issues in this factor are controlled by senior managers and reflect relevant management policies [20]. That is to say, managers’ behaviors will be subject to management regulations and rules [89]. From workers’ family environments and the work environment, family members and colleagues are an important social reference when there are risks in a highly dangerous working environment [90,91]. Therefore, in this paper, social influencing factors mainly include unit training situation, related rules and regulations, the manager’s attitude, group effect, and the family environment. As stated above, the following research hypothesis are thus formulated:

**Hypothesis** **3a** **(H3a).** *Unit training has a positive impact on occupational health risk perception of construction workers.*

**Hypothesis** **3b** **(H3b).** *Related rules and regulations have positive impacts on occupational health risk perception of construction workers.*

**Hypothesis** **3c** **(H3c).** *Manager attitude has a positive impact on occupational health risk perceptions of construction workers.*

**Hypothesis** **3d** **(H3d).** *Group effect has a positive impact on occupational health risk perception of construction workers.*

**Hypothesis** **3e** **(H3e).** *Family environment has a positive impact on occupational health risk perception of construction workers.*

In the theory of health protection behavior, it is believed that the behavior of reducing risk partly depends on the level and likelihood of perception of health risks and expectations [92,93]. Therefore, Xu and Tan [94] argued that risk perception is a positive predictor of adaptive behavior and an insignificant predictor of mitigation behavior. Joffre [95], when analyzing the coexistence of different types of shrimp farms in the same landscape in the Mekong Delta in Vietnam, found that market risk perception has a significant impact on risk management strategies. Risk perception is a key predictor of risk management strategies. Therefore, the degree of perception and perception of construction workers to various objective risks existing in the external environment may also have a certain impact on their responses to deal with risks. Based on the above discussion, Hypothesis 4 is put forward as follows:

**Hypothesis** **4** **(H4).** *The occupational health risk perception of construction workers has a positive effect on their coping behavior.*

During the process of literature review, it was found that risk perception plays a mediating role. For example, Lim [96] found that the mediation effect of risk perception is significant in the continuous positive relationship between financial knowledge and financial behavior investment intention. In addition, Boo [97] pointed out that the understanding of body mass index (BMI) and weight perception has a clear correlation with efforts of weight loss. People who are overweight or obese but fail to know this themselves are less likely to try to lose weight. Those who underestimate their weight need to understand the definition of healthy weight. Therefore, weight perception is indeed a partial mediator between the understanding of BMI and weight loss. Hong [98] proved that risk perception plays a mediating role between media exposure and emergency preparedness cooperation. Similarly, the risk perception of the construction workers for interior decoration may also be a mediator between individual characteristics, personal knowledge, social influence factors, and coping behavior. As stated above, Hypothesis 5a–5c are proposed as follows:

**Hypothesis** **5a** **(H5a).** *Construction workers’ occupational health risk perceptions play mediating roles between their individual characteristics and coping behaviors.*

**Hypothesis** **5b** **(H5b).** *Construction workers’ occupational health risk perceptions play mediating roles between their personal knowledge and coping behaviors.*

**Hypothesis** **5c** **(H5c).** *Construction workers’ occupational health risk perceptions play mediating roles between social influence factors and coping behaviors.*

**Figure 1:**
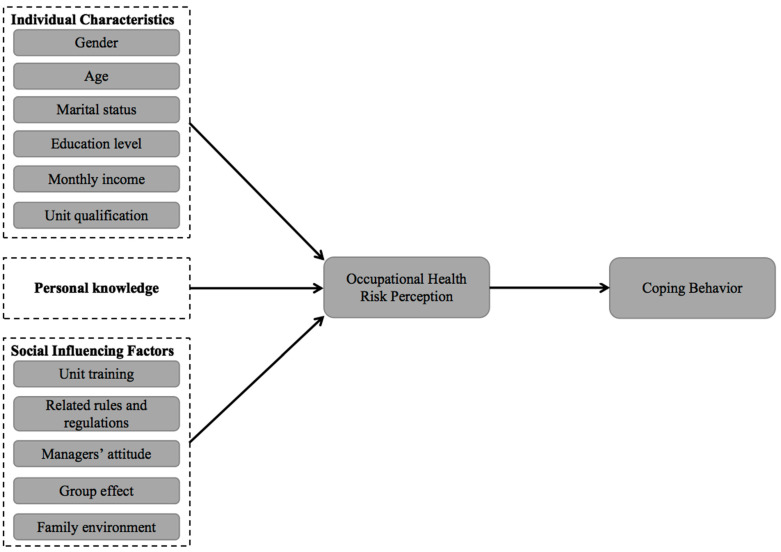
The model of occupational health risk perception–coping behavior.

Based on the above hypothesis, a model is established regarding construction workers’ occupational health risk perceptions and coping behaviors towards pollutants (Figure 1). 

## 4. Methodology

**Figure 2:**
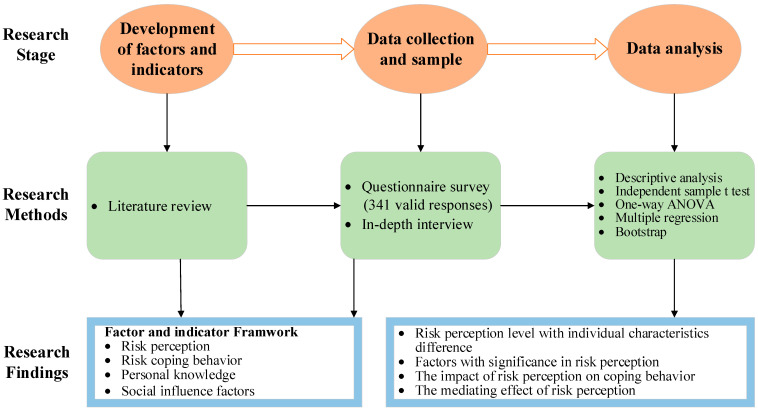
The methodology framework in the study.

The methodology adopted to achieve the research objectives consisted of a literature review, questionnaire survey, and in-depth interview, as illustrated in Figure 2.

First, the indicators of construction workers’ occupational health risk perceptions and coping behaviors were initially collected through literature retrieval in various databases (such as Web of Science, Science Direct, Engineering Village (EI)), detailed in Table 1.

The comprehensive literature review also supported the development of a survey questionnaire. The final questionnaire included five parts: (1) demographic information of the construction workers; (2) occupational health risk perception of construction workers; (3) occupational health risk coping behaviors of construction workers; (4) respondents’ basic knowledge of decoration pollution; and (5) respondents’ viewpoints on social influence factors. A pilot study in the surrounding communities was conducted to validate the accuracy and completeness of the questionnaire script. Responses were measured on a five-point Likert scale (1 represents strongly disagree and 5 represents strongly agree, or 1 represents totally inconsistent and 5 represents totally consistent). In addition to measuring the occupational health risk perception level and coping behavior level of the construction workers, the scores of all variables in the individual scale were added up to calculate the average value, which was taken as the total index. It was generally considered that workers had relatively good risk perception level and coping behavior level when the score was higher than 3, since the five-point Likert scale was used, and 3 represents the middle level [127,128]. 

The questionnaire targeted the construction workers for interior decoration. The survey was carried out in July and August 2019, mainly in residential districts in Jiangning District and Pukou District of Nanjing, China, where there are a large number of decoration projects due to the large transaction volume of new real estate and second-hand housing. The criteria for selecting samples ensured that all types of workers were involved and that there was an average number of each type of worker. Considering the limited educational background of construction workers and the difficulty of completing the questionnaire independently, the survey was conducted in a face-to-face manner. For those with difficulties completing it independently, the professionally trained investigators assisted on site. The investigators explained the unknown items of the research object and ensured the consistency of the explanatory contents of each item. Consequently, in-depth interviews were conducted to everyone who filled out the questionnaire to confirm the reasons for their choices. They were administered to a total of 341 workers engaged in interior decoration, and all of their responses were valid, resulting in an effective response rate of 100%. Table 2 profiles the demographic information of the respondents. It can be observed that over 94 percent respondents were male, and 90 percent of respondents were married. Nearly three quarters were in the age group between 30 and 50, and more than half of respondents were generally not highly educated (with the education of middle school), while the number of construction workers with education levels of junior college was relatively small (only 12.3%). From the perspective of income level, the incomes of construction workers surveyed were mostly concentrated in the RMB 5000–9000 range. Moreover, nearly 40% of the respondents did not know the qualifications of their own construction units.

Statistical analysis was performed using the SPSS software package version 22.0 (IBM, Armonk, NY, USA). First, the validity and reliability of each variable of the measurement model were conducted. The reliability test values of all the scales and the questionnaire were greater than 0.6, indicating that the scale of coping behavior was acceptable, and the overall questionnaire and the other three scales had good reliability. In addition, the results showed that the overall Kaiser-Meyer-Olkin (KMO) value was 0.889, which was above the recommended value of 0.60 [129]. The Bartlett spherical test was significant (*p* < 0.05). Both indicates that the questionnaire was suitable for conducting factor analysis (refer to Table 3 for details). Moreover, descriptive analysis was performed to reveal current occupational health risk perception levels and coping behavior levels of construction workers.

Second, to check whether there were significant differences for respondents’ occupational health risk perception levels in terms of their demographics, one-way analysis of variance (ANOVA) and independent sample t-tests were adopted. To test the potential difference of means between two groups of samples, independent sample t-tests were used. Generally speaking, if the data meets the uniform variance, we can perform an independent sample t-test. Otherwise, the t’-test can be used instead. To test the potential difference of means of more than two groups of samples, one-way ANOVA is used. For one-way ANOVA, if the assumption of homogeneity of variance is not satisfied, a corrected one-way analysis of variance must be used, such as Welch’s analysis of variance. The data samples of each group were tested before the analysis of variance, and the results showed that the samples met the premise of independence and obeyed a normal distribution, and the variance between each group was homogeneous. 

Third, to examine whether the identified factors significantly influenced the occupational health risk perception and risk coping behaviors, respectively, based on the model constructed, multiple regression was used, since it is more effective to predict dependent variables by the optimal combination of multiple independent variables than to use only one independent variable for prediction.

Last, to test the mediating role of risk perception, bootstrapping was employed. Regression analysis was performed using PROCESS Model 4 developed by Hayes [130]. Specifically, three regression equations were run to verify the following conditions: (a) independent variable (X) affects dependent variable (Y); (b) independent variable (X) affects mediator (M); and (c) mediator (M) affects dependent variable (Y) [131]. Fully mediating associations were tested when the direct effect of X on Y was not statistically significant after controlling for the effect of M; partial mediation was tested when controlling for M, and the direct effect of X on Y was significant [131]. A bootstrapping procedure with n = 5000 bootstrap re-samples was used to evaluate the indirect effects [132,133,134]. An indirect effect was tested significant if the 95% bias corrected and accelerated bootstrapped confidence interval (Boot95%CI) excluded zero.

## 5. Results

### 5.1. Current Risk Perception Level and Coping Behavior Level of Construction Workers

Table 4 presents the current occupational health risk perception level of construction workers. From the table, it can be seen that the overall average value of risk perception of construction workers was 4.03, which was at a good level (above 4), and the standard deviation was 0.7, leading to the conclusion that the occupational health risk perception of respondents was relatively consistent. The decorating workers had a strong risk perception of “dust exposure leads to pneumoconiosis”, with the mean value of 4.29. Relatively speaking, the respondents’ risk perception of “more exercise and less smoking can reduce the probability of illness” was low.

Table 5 shows the current situation of construction workers’ coping behavior to occupational health risks. As can be seen from the table, the overall mean value of the coping behaviors of the construction workers was 3.15, and the standard deviation was 0.7. As a result, the coping behaviors of the construction workers was relatively consistent, and the fluctuation range was not obvious. Nevertheless, compared with occupational health risk perception of construction workers on occupational health risk, their coping behavior was not ideal. In addition, of all six variables, most workers chose “maintain ventilation” to deal with occupational risks, showing that maintaining ventilation has become a necessary protective measure for construction workers in the process of construction work. Moreover, the majority of respondents did not agree that the unit would “pay insurance and carry out physical examination”, as the average was only 2.25, and the standard deviation was up to 1.24.

### 5.2. Hypothetical Test

#### 5.2.1. Differences Analysis of Individual Characteristics in Occupational Health Risk Perception

Results of the differences of the respondents’ individual characteristics in occupational health risk perceptions are shown in Table 6. In terms of occupational health risk perception, gender, age, education level, monthly income, and qualifications of the unit resulted in significant differences (*p* < 0.05) among construction workers for interior decoration—a support for H1a, H1b, H1d, H1e and H1f. Marital status, however, did not affect the level of construction workers’ risk perceptions (*p* > 0.05). Therefore, H1c was not verified. Specifically, the risk perception level of male construction workers was lower than that of female ones. The occupational health risk perception of construction workers under 30 years old and over 50 years old was at a relatively good level, and the level of occupational health risk perception generally increased with rising incomes and improvement of education levels. In addition, the level of occupational health risk perception of construction workers working in different qualification units was quite different. Moreover, the lower the qualification level of the units, the worse the occupational health risk perception level of construction workers. 

#### 5.2.2. The Impact of Personal Knowledge on Risk Perception

The analysis results of construction workers’ personal knowledge and their occupational health risk perceptions are shown in Table 7. It can be seen that personal knowledge had a significant effect on the decoration pollution risk perception (*p* < 0.001). Moreover, the non-standardized coefficients and standardized coefficients of the equation were 2.069 and 0.519, respectively (both greater than 0), indicating that personal knowledge had a positive impact on risk perception, i.e., the more knowledge the construction worker has, the higher their risk perception level will be. H2 is thus supported.

#### 5.2.3. The Impact of Social Influencing Factors on Risk Perception

Table 8 showed that when exploring the relationship between social influencing factors and risk perception, the group effect was the only factor that had a significant impact on occupational health risk perception (*p* < 0.05), and H3d was therefore tested. However, no statistically significant impact was tested in unit training, related rules and regulations, manager’s attitude, and family environment (*p* > 0.05). Accordingly, H3a, H3b, H3c and H3e were not supported. To some extent, the behaviors of the group are affected by the surrounding environment, and the ideas and behaviors of the surrounding people affect the level of occupational health risk perception of the construction workers.

#### 5.2.4. The Impact of Risk Perception on Coping Behavior

Based on the above discussion and analysis, the internal relationship of occupational health risk perception and coping behaviors of construction workers for interior decoration was further explored. The analysis results are shown in Table 9. The occupational health risk perception of construction workers had a significant impact on their coping behavior (*p* < 0.001). On the other hand, as the non-standardized coefficient and the standardized coefficient were both positive, it can be explained that under the decoration pollution risk, the coping behaviors of the construction workers were positively related to their risk perceptions, and the decoration pollution risk perceptions had a positive impact on the response behaviors. Thus, H4 is supported.

#### 5.2.5. Mediating Effect of Risk Perception

As can be seen from Table 10, gender, age, and marital status had no impact on copying behavior. Therefore, in the relationship of the above factors and coping behaviors, the mediating effects of occupational health risk perception did not exist. Looking at the other factors that had significant impacts on coping behaviors, from the value of Boot95%CI in the table, the mediating effects of occupational health risk perception on the linkages between education level, monthly income, and personal knowledge, and coping behaviors were significant (exclude zero). Hence, H5a was partially reinforced. Support was also strong for H5b. The results of the mediating effect are listed in Table 11. Among the three paths, as a mediating variable, occupational health risk perception had the greatest effect on the relationship between personal knowledge and coping behavior (indirect effect: 0.543, up to 52.98%), followed by the effect on the relationship between education level and coping behavior (indirect effect: 0.064, accounted for 31.68%). The relationship of monthly income and copying behavior was the least affected by the occupational health risk perception (indirect effect: 0.048, accounted for 30.38%). In addition to the factors mentioned above, the effect of other factors on coping behavior was not affected by occupational health risk perception; they all affected coping behaviors directly (*p* < 0.05). Thus, H5c is not supported.

## 6. Discussion

### 6.1. Risk Perception Level and Coping Behavior Level of Construction Workers

Based on the above analysis, the level of occupational health risk perception of construction workers is good, which may be related to the education and supervision of relevant government departments and enterprises in occupational health [135,136]. Construction workers for interior decoration, however, have poor risk perception of prevention of decoration pollution. Therefore, the enterprise should be equipped with corresponding protective equipment [10] and strengthen the management of artificial control [10]. Moreover, experts and scholars in related areas ought to strive for technological breakthroughs [137]. Moreover, the government is suggested to play a supervisory role in enterprises’ management of employees’ occupational health [138].

However, compared with the level of perception of construction workers on occupational health risk, the level of their coping behavior is not very ideal, which may be ascribed to the lack of decoration pollution protection equipment [139], the shortage of funds [140], and the lack of attention from the units and relevant government departments [135]. Therefore, in addition to the routine management such as the supervision of construction progress and project quality, the occupational health of construction workers should be considered in units’ daily management [141], and health protection is required to be provided as much as possible [142].

### 6.2. Differences in Perception of Occupational Health Risk

Through hypothesis testing, we found that there is no difference in the occupational health risk perception level of construction workers in terms of marital status. Gender, age, education level, monthly income, and unit qualification can cause differences in the occupational health risk perception level of construction workers. Female workers’ occupational health risk perception levels are higher than those of male workers. Thus, the occupational health risk perception of male workers should be strengthened. The risk perception level of construction workers between 30 and 50 years old is relatively low. Construction workers in the age group of 30–50 years old usually feel that they are in good health and not prone to illness, and thus have poor awareness of prevention. Therefore, education and training should be more emphasized towards workers between the ages of 30 and 50. They are mainstays in the construction worker team [143]. It is very important to strengthen their awareness of self-protection. 

When it comes to education level, the higher it is, the better the occupational health risk perception. The reason might be that construction workers with higher education have broader knowledge of the decoration pollutants and their harms to people, as well as the stronger ability to understand [144,145]. In contrast, construction workers with junior high school or below are lacking in relevant knowledge and ability, requiring the relevant units not to simply adopt centralized training and preaching, but to establish the health risk perception of construction workers in a subtle way by holding lectures as many times as possible [146].

As the income level is concerned, the higher it is, the higher the risk perception of the construction workers is. This may be due to the fact that the low-income group first considers meeting the most basic living needs and has no time to take care of higher-level needs (health) [147,148]. In addition to meeting the most basic living needs, upper-middle-income workers still have the ability to pursue higher level needs, and their attention to the hazards in the construction process will increase accordingly [149]. Therefore, the government and enterprises should improve the welfare guarantee system and provide appropriate subsidies [150], especially for workers whose monthly income is less than RMB 5000, to ensure that they have a certain balance in addition to meeting living needs and guide them to establish a better sense of risk. 

For the qualifications of construction companies, the higher the qualification level of construction companies is, the higher the level of risk awareness of their workers might be. This may be related to the management ability [151,152], the management techniques in the occupational health of construction units with different qualifications [153], as well as the education of risks [154] and relevant inspections of the units [155,156]. Therefore, the enterprises with level A and level B construction qualification do relatively well at present. Enterprises with level C construction qualification and those without qualification need further efforts to meet the standards. 

### 6.3. Influencing Factors on Occupational Health Risk Perception

When testing whether there are relationship influences in the model, it is found that personal knowledge has a significant influence on construction workers’ occupational health risk perception. The higher the degree of personal knowledge, the higher the level of risk perception of the construction workers. Hence, it is necessary for enterprises and governments to increase health and safety training for construction workers, including pre-job training and regular training at work [140,157,158]. If necessary, training methods could be improved [159], such as adding case teaching to make workers have a more intuitive sense of risk existing in the construction site.

Furthermore, considering the five aspects of social influencing factors, the results showed that only group effects have a significant impact on construction workers’ occupational health risk perceptions. If the surrounding workers can wear corresponding protective equipment in accordance with regulations and take certain measures to prevent the potential risks during construction, the construction worker will be affected by them. It is suggested that enterprises should establish a group responsibility system for evaluating, rewarding, and punishing the entire group of protection work [160]. As a result, group members can affect and monitor each other. Contrary to previous research, unit training, related rules and regulations, and managers’ attitudes have nothing to do with construction workers’ occupational health risk perception. The construction industry is characterized by a large number of migrant workers with low educational backgrounds, high construction requirements, and high mobility of workplaces [161]. Therefore, the training currently implemented in the construction industry is still focused on construction skills, and most of these trainings are carried out directly during the construction process [162]. This result is also consistent with the content of the interview. Construction workers have almost never participated in occupational health training, and they do not even know whether their enterprises have occupational health management regulations and related protective measures. Regulations alone cannot reduce risks unless workers and managers take positive actions to incorporate these rules into their daily activities [19]. However, from in-depth interviews, the managers that construction workers interact with are supervisors who are only concerned about construction progress and quality, and hardly mention occupational health issues. This also explains why managers’ attitudes were not confirmed as an important factor affecting construction workers’ risk perceptions in this study. Only formulation of occupational health risk management regulations by enterprises cannot guarantee the occupational health of construction workers. As Langford et al. (2020) said, if workers think that enterprises and managers care about their personal safety, then they are more willing to cooperate. Collective efforts play an important role in safety improvement, which also applies to occupational health management [20]. Therefore, health training centered on construction workers and supplemented by managers should be emphasized [163], including regular lectures to update occupational health-related information or distribute occupational health learning materials to construction workers, etc. [164]. Moreover, the interaction between managers and workers also plays a pivotal role in safeguarding workers’ occupational health, because to a certain extent, it quickly adjusts workers’ behaviors in response to occupational health risks [165]. This action-centered reflection forces workers to infer the relationship between risk and behavior, so as to formulate more effective strategies to deal with occupational health risks.

### 6.4. The Mediating Effect of Occupational Health Risk Perception

Construction workers’ occupational health risk perception plays a mediating role in the relationship of their education levels, personal knowledge, monthly incomes, and coping behaviors. On the one hand, construction workers’ personal knowledge, education levels, and monthly incomes have a direct positive effect on their coping behaviors, i.e., the higher the construction workers’ personal knowledge, education levels, and monthly incomes are, the better their coping behaviors will be. On the other hand, personal knowledge, education level, and monthly income have an indirect positive effect on behaviors through the mediator-occupational health risk perception, i.e., if the construction workers have better knowledge of construction risks, higher education levels, and higher monthly incomes, the levels of their occupational health risk perception will be higher, which will affect their coping behaviors, so that construction workers ought to take more active coping behaviors. Moreover, occupational health risk perception has the greatest effect on the relationship between personal knowledge and coping behavior. Therefore, in order to take effective and scientific measures to improve construction workers’ abilities to deal with the risks of decoration pollutants, the enterprises need to start by improving workers’ knowledge of construction risks, ensuring the incomes of construction workers, and organizing them to learn the sources, hazards, and countermeasures of various risks. Through this, workers are expected to indirectly improve their cognitive levels of the risks generated during their working process and make positive, reasonable, and correct coping behaviors.

## 7. Conclusions

This paper took construction workers for interior decoration as the research subjects, targeting their occupational health risk perceptions and coping behaviors, and analyzing their occupational health risk perception levels and coping behavior levels. The overall risk perception of construction workers is at a good level, while their coping behaviors are generally poor. Moreover, the results of differential analysis showed that at this stage, construction workers of different genders, ages, education levels, monthly incomes, and unit qualifications have different levels of occupational health risk perception.

Moreover, the influencing factors of occupational health risk perception level and coping behavior level of construction workers were tested based on the constructed model. The individual occupational health risk perception level of construction workers is affected by group effect and personal knowledge. Therefore, the knowledge of construction workers and the group effect of the construction site should be noted. Furthermore, construction workers’ occupational health risk perceptions significantly affect their coping behaviors. On this basis, taking the occupational health risk perception as the mediating variable, the internal relationship of the occupational health risk perception and coping behavior was deeply discussed. Construction workers’ occupational health risk perceptions plays a partial mediating role between workers’ personal knowledge, education levels, monthly incomes, and coping behaviors. Hence, the occupational health perception of construction workers is worthy of everyone’s attention. Finally, some targeted suggestions were proposed based on the results of the case analysis.

Findings of this research offer both theoretical and practical implications. From a theoretical perspective, findings are expected to complement the research in the field of risk perception, broaden the research perspective in the field of occupational health, and advance the existing body of knowledge. At the same time, the test of the mediating role of risk perception is helpful to strengthen the research in the related fields of civil engineering in the future, i.e., in different situations and for different research objects, risk perception can be considered as an important variable to measure research problems. In terms of the implications for practitioners, the research findings can make construction workers clearly understand that their current protective measures against occupational health risks are far from enough. Simultaneously, based on the analysis of influencing factors, this study proposed a few practical measures and suggestions from the perspectives of individuals, enterprises, and government departments, so as to strengthen their understandings and perceptions of occupational health risks, and thereby alter their coping behaviors. It aims to form a system of tripartite cooperation of enterprise–manager–workers under the leadership of the government to reduce workers’ occupational health risks. Moreover, it can also provide guidance for other types of construction workers to prevent occupational health risks.

While this study exposed a rather neglected area of environmental health and safety issues, there are unfortunately some limitations. The present survey was restrictedly conducted in Nanjing, China. In addition, only construction workers for interior decoration were investigated. Therefore, we propose that further research should collect more samples from different regions and different types of construction workers for comparative analysis. In addition, due to the limitations of psychology related knowledge, there are some defects in the setting of questions in the questionnaire, which need to be further improved and refined. 

## Figures and Tables

**Table 1:** Specific indicators of construction workers’ risk perceptions and risk coping behaviors on occupational health risk.

Categories	Indicator	Literature Sources
Risk Perception	Hazardous substances are generated	Li et al. (2019) [99]; Shim et al. (2017) [100]
	Dust exposure leads to pneumoconiosis	Chen et al. (2019) [33]; Wu et al. (2016) [101]
	Formaldehyde causes chronic respiratory diseases	Zhuo (2018) [102]; Xia et al. (2012) [103]
	Inhalation of irritant gas causes headache	Chong et al. (2018) [104]; Cui et al. (2020) [105]
	Materials with less pollution reduce the probability of illness	Cincinelli et al. (2017) [106]; Yan et al. (2017) [107]
	Protective equipment (dust masks, gas masks, etc.) reduces harm caused by pollution	Li et al. (2017) [108]; Lette et al. (2018) [109]; Tadesse et al. (2016) [110]
	Ventilation reduces harm of formaldehyde, benzene, and dust to the body	Weidman et al. (2016) [111]; Chen et al. (2020) [112]
	Decoration pollution can be prevented	Li et al. (2016) [113]; Liqun et al. (2011) [114]
	More exercise and less smoking can reduce the probability of illness	Tadesse et al. (2016) [110]; Sawicki et al. (2020) [115]; Vitharana et al. (2019) [116]
Coping Behavior	Maintain ventilation	Yan (2017) [107]; Chang et al. (2017) [117]
	Wear dust mask	Shepherd et al. (2010) [118]; Feng et al. (2013) [119]
	Wear labor protection shoes	Goto et al. (2017) [120]; Suo et al. (2017) [121]
	Use other personal protective equipment	Kohlman et al. (2014) [122]; Li et al. (2019) [99]
	Avoid eating and resting on site	Yi et al. (2016) [123]; Brahmachary et al. (2018) [124]
	Pay insurance and carry out physical examination	Kim et al. (2010) [125]; Adsul et al. (2011) [126]

**Table 2:** Demographic information of survey respondents.

Variable	Classification	NO.	Proportion	Variable	Classification	NO.	Proportion
Gender	Male	321	94.10%	Education level	Junior middle school or below	188	55.10%
	Female	20	5.90%		Technical secondary school or high school	111	32.60%
Marital status	Married	307	90.00%		Junior college or above	42	12.30%
	Single	34	10.00%	Monthly income (RMB)	≤5000	47	13.80%
Trades	Carpenters	76	22.30%		5000–7000	164	48.10%
	Bricklayers	70	20.50%		7000–9000	109	32.00%
	Electricians	70	20.50%		≥9000	21	6.20%
	Plumbers	45	13.20%	Unit qualification	Level A	79	23.20%
	Painters	66	19.40%		Level B	40	11.70%
	Other	14	4.10%		Level C	23	6.70%
Age	≤30	35	10.30%		No qualification	78	22.90%
	30~50	243	71.30%		Unclear	121	35.50%
	≥50	63	18.50%				

Note: (1) respondents (total = 341); (2) the qualification of interior decoration construction units is divided into three levels: A, B and C. Units with level A qualification are the best, followed by level B and level C.

**Table 3:** Validity and reliability analysis.

Variable	Item	Factors				Cronbach’s α ^a^
		1	2	3	4	
Occupational health risk perception	B1		0.682			0.872
	B2		0.767			
	B3		0.754			
	B4		0.707			
	B5		0.467			
	B6		0.628			
	B7		0.602			
	B8		0.523			
	B9		0.561			
Coping behaviors	C1				0.277	0.668
	C2				0.531	
	C3				0.531	
	C4				0.699	
	C5				0.347	
	C6				0.669	
Personal knowledge	D1			0.699		0.834
	D2			0.854		
	D3			0.854		
	D4			0.744		
	D5			0.294		
	D6			0.688		
	D7			0.675		
Social influence factors	E1	0.877				0.936
	E2	0.882				
	E3	0.721				
	E4	0.884				
	E5	0.712				
	E6	0.737				
	E7	0.791				
	E8	0.795				
	E9	0.851				
	E10	0.493				
	E11	0.688				
KMO = 0.889, Bartlett Χ^2^(df) = 6298.903(528) ***						

Note: ^a^ Overall Cronbach’s α = 0.879. *** *p* < 0.001.

**Table 4:** Perception of construction workers to occupational health risks.

Variable	Mean ± SD
Hazardous substances are generated	4.28 ± 0.88
Dust exposure leads to pneumoconiosis	4.29 ± 0.86
Formaldehyde causes chronic respiratory diseases	4.19 ± 0.93
Inhalation of irritant gas causes headache	3.92 ± 1.00
Materials with less pollution reduce the probability of illness	3.93 ± 1.14
Protective equipment (dust masks, gas masks, etc.) reduces harm caused by pollution	3.86 ± 1.00
Ventilation reduces harm of formaldehyde, benzene, and dust to the body	4.10 ± 0.92
construction pollution can be prevented	4.05 ± 0.97
More exercise and less smoking can reduce the probability of illness	3.77 ± 1.24
Risk perception	4.03 ± 0.70

**Table 5:** Coping behavior of construction workers to occupational health risks.

Variable	Mean ± SD
Maintain ventilation	3.97 ± 0.93
Wear dust mask	3.33 ± 1.19
Wear labor protection shoes	3.53 ± 1.13
Use other personal protective equipment	2.40 ± 1.23
Avoid eating and resting on site	3.36 ± 1.16
Pay insurance and carry out physical examination	2.25 ± 1.24
Coping behavior	3.15 ± 0.70

**Table 6:** Risk perception differences among construction workers.

Variable	Classification	Mean ± SD	Levene’s Test (Sig)	*p*-Value	*F*-Value	*T*-Value
Gender	Male	4.02 ± 0.71	0.051	0.035	/	−2.116
	Female	4.36 ± 0.54				
Age	≤30	4.32 ± 0.72	0.274	0.007	5.083	/
	30–50	3.97 ± 0.69				
	≥50	4.17 ± 0.71				
Marital status	Married	4.05 ± 0.72	0.044	0.194		1.319
	Single	3.91 ± 0.55				
Education level	Junior middle school or below	3.87 ± 0.76	0.000	0.000	15.109	/
	Technical secondary school or high school	4.21 ± 0.60				
	Junior college or above	4.32 ± 0.48				
Monthly income (RMB)	<5000	3.63 ± 0.95	0.000	0.033	3.055	/
	5000–7000	4.04 ± 0.69				
	7000–9000	4.10 ± 0.62				
	>9000	4.23 ± 0.67				
Unit qualification	Level A	4.20 ± 0.64	0.000	0.002	4.636	/
	Level B	4.03 ± 0.61				
	Level C	3.86 ± 0.52				
	No qualification	3.76 ± 0.85				
	Unclear	4.15 ± 0.64				

**Table 7:** The results of the impacts of personal knowledge on risk perception.

Model	Non-Standardized Coefficient		Standardized Coefficient	*t*-Value	*p*
	B	SE	β		
Constant	2.858	0.111		25.849	0.000
personal knowledge	2.069	0.185	0.519	11.182	0.000

Dependent variable: risk perception.

**Table 8:** The results of the impacts of social influencing factors on risk perception.

Model	Standardized Coefficient	*t*-Value	*p*
Constant		30.985	0.000
Unit training	−0.090	−0.944	0.346
Related rules and regulations	−0.093	−0.823	0.411
Manager’s attitude	−0.161	−1.784	0.075
Group effect	0.268	3.201	**0.002 ^a^**
Family environment	0.034	0.466	0.642

Dependent variable: coping behavior. ^a^ Bold represents a significant correlation.

**Table 9:** The results of the impacts of risk perception on coping behavior.

Model	Non-Standardized Coefficient		Standardized Coefficient	*t*-Value	*p*
	B	SE	β		
Constant	1.822	0.212		8.578	0.000
Risk perception	0.325	0.052	0.323	6.278	0.000

Dependent variable: coping behavior.

**Table 10:** Mediating effects of occupational health risk perception.

Independent Variable (Xi_1_)	Model 1 (Y: Coping Behavior)		Model 2 (Y: Risk Perception)		Model 3 (Y: Coping Behavior)				Boot95%CI
	Xi_1_		Xi_1_		Xi_1_		Xi_2_ = Risk Perception		
	β	*t*	β	*t*	β	*t*	β	*t*	
Gender	0.35	1.507^0.133^							
Age	−0.07	−1.233^0.222^							
Marital status	0.24	1.335^0.183^							
Education level	0.22	4.188 ***	0.25	4.69 ***	0.15	2.883 **	0.29	5.437 ***	(0.036, 0.098)
Monthly income	0.18	3.283 **	0.18	3.287 **	0.12	2.364 *	0.30	5.806 ***	(0.016, 0.081)
Unit qualification	−0.25	−4.757 ***	−0.05	−0.974^0.331^	−0.23	−4.679 ***	0.31	6.212 ***	(−0.021, 0.010)
Personal knowledge	0.26	4.86 ***	0.52	11.18 ***	0.12	2.01 *	0.26	4.35 ***	(0.315, 0.789)
Unit training	0.33	6.472 ***	0.07	−1.353^0.177^	0.36	7.468 ***	0.35	7.295 ***	(−0.034, 0.005)
Related rules and regulations	0.39	7.903 ***	−0.09	−1.592^0.112^	0.43	9.207 ***	0.36	7.778 ***	(−0.044, 0.003)
Manager’s attitude	0.34	6.651 ***	−0.11	−1.947^0.052^	0.38	7.952 ***	0.36	7.628 ***	(−0.044, 0.001)
Group effect	0.38	7.542 ***	0.06	1.130^0.260^	0.36	7.561 ***	0.30	6.302 ***	(−0.009, 0.037)
Family environment	0.35	6.866 ***	0.01	0.168^0.867^	0.35	7.232 ***	0.32	6.670 **	(−0.018, 0.022)

Note: * *p* < 0.05, ** *p* < 0.01, *** *p* < 0.001.

**Table 11:** Mediating effect test of Bootstrap.

Hypothesized Paths	Effect Type	Effect Value	BootSE	Boot95%CI	Percentage
Education level→risk perception→coping behavior	Total effect	0.202	0.058	(0.122, 0.352)	
	Direct effect	0.138	0.056	(0.031, 0.250)	68.32%
	Indirect effect	0.064	0.016	(0.036, 0.098)	31.68%
Monthly income→risk perception→coping behavior	Total effect	0.158	0.052	(0.053, 0.259)	
	Direct effect	0.110	0.049	(0.015, 0.205)	69.62%
	Indirect effect	0.048	0.017	(0.016, 0.081)	30.38%
Personal knowledge→risk perception→coping behavior	Total effect	1.025	0.192	(0.654, 1.406)	
	Direct effect	0.482	0.225	(0.050, 0.929)	47.02%
	Indirect effect	0.543	0.122	(0.315, 0.789)	52.98%

## Data Availability

The data presented in this study are available on request from the corresponding author. The data are not publicly available due to some subsequent studies that rely on the data obtained from the survey.
